# Interrater Reliability of a Modified Bronchoscopy Scoring Tool in Children With Cystic Fibrosis

**DOI:** 10.1002/ppul.71452

**Published:** 2025-12-30

**Authors:** Alexandra Bosetti, Srdjan Micic, Andreas Hector, Christian Bieli, Elias Seidl, Alexander Moeller

**Affiliations:** ^1^ Department of Respiratory Medicine University Children's Hospital Zurich Zürich Switzerland; ^2^ Division of Paediatric Pulmonology, Cantonal Hospital of Winterthur Winterthur Switzerland; ^3^ Division of Paediatric Pulmonology, Cantonal Hospital of Aarau Aarau Switzerland

**Keywords:** bronchoscopy, children, cystic fibrosis, inter‐rater reliability, scoring tool

## Abstract

**Background:**

Flexible bronchoscopy (FB) is widely used in the management of children with Cystic Fibrosis (CF) to visualize airway abnormalities, assess inflammation and detect infection. While previous scoring systems have been proposed to quantify visual airway findings in general pediatric populations, no standardized tool exists for assessing airway inflammation specific to CF.

**Methods:**

We modified the previously proposed pediatric bronchoscopy scoring tool by adding four features relevant to CF pathology: mucus plugging, secretion viscosity, bleeding, and vascular drawing (abnormal or enhanced visualization of airway mucosal vessels, reflecting neovascular remodeling associated with inflammation). Eighty bronchoscopy recordings (50 CF, 30 non‐CF) were retrospectively scored by four raters blinded to the clinical information, and ten visual features were assessed: six from the previously proposed score (secretion amount and color, mucosal edema, erythema, ridging, and pallor) and the four CF‐specific additions. Inter‐rater reliability was assessed using Gwet's AC2 coefficient.

**Results:**

Agreement between raters varied across features. Mucus plugging (AC2 = 0.94) and bleeding (0.80), both CF‐specific, were among the most reliably scored features, while secretion viscosity (0.47) and vascular drawing (0.50) showed the lowest agreement. The expanded score demonstrated comparable or improved reliability for overlapping features from earlier scoring systems.

**Conclusion:**

The modified bronchoscopy score demonstrated moderate to excellent inter‐rater reliability and added clinically relevant features specific to CF. It may serve as a standardized method to assess bronchoscopy for pediatric CF lung disease, although further validation is needed for features with lower inter‐rater reliability.

## 1 Introduction

Cystic Fibrosis (CF) is a genetic, multi‐organ disorder genetic disorder characterized by impaired mucociliary clearance, airway inflammation and infection with opportunistic pathogens [1, 2, 3]. Flexible fiberoptic bronchoscopy (FB) is a widely used diagnostic tool in pediatric pulmonology, offering direct visualization of airway abnormalities and mucosal changes. In children with CF, FB is particularly valuable for assessing features such as edema, amount and consistency of mucus, mucus plugging, and abnormal vascular patterns, which reflect the underlying inflammation and infection associated with CF lung disease [4, 5]. It provides additional diagnostic insight when imaging and clinical findings are inconclusive, and supports targeted management decisions, especially in children not able to expectorate sputum for microbiology testing. However, visual assessment during FB remains largely subjective and lacks standardized criteria for quantifying inflammatory changes. A reliable and reproducible scoring system enables objective evaluation of airway inflammation, facilitates longitudinal assessment of disease progression or response to therapy, and supports comparison of bronchoscopic findings across studies and disease entities. In a first attempt to standardize visual assessment with FB, Thompson et al. introduced the qualitative bronchitis index for adults using visualization of edema, erythema, secretions, and friability during FB in adult patients with chronic bronchitis [6]. The results suggested that the score was a potentially reliable and reproducible tool for grading visual changes of airway disease in a semi‐quantitative manner. Later, Thomas et al. adapted this score for use in children by modifying the parameters to include secretion color, mucosal ridging, and pallor, while excluding friability [7]. Their study demonstrated feasibility and adequate inter‐rater agreement for several visual features in a general pediatric population but were not tailored to CF‐specific airway pathology inflammation (i.e. mucus plugging, secretion viscosity, bleeding, and vascular drawing) [2]. Therefore, there remains a need to develop and validate a CF‐adapted bronchoscopy scoring tool that can reliably capture disease‐relevant features and promote standardized assessment in both clinical and research settings.

The aim of our study was to expand the bronchoscopic scoring system proposed by Thomas et al. by adding visual features specific for CF airways disease. We assessed the inter‐rater reliability of this modified bronchoscopy scoring tool in a cohort of children undergoing FB and compared the agreement levels of overlapping features with those reported in the original study to evaluate consistency.

## 2 Methods

### 2.1 Study Design

This retrospective study analyzed digital recordings of flexible FB procedures performed on children at the University Children's Hospital Zurich between 2018 and 2021. The study included 80 randomly selected recordings, consisting of 50 children with CF and 30 non‐CF patients. The videos had to include five predefined lobes: right upper, right middle, right lower, left upper and left lower lobe. FB recordings were performed using flexible Olympus or Pentax bronchoscopes and stored as AVI files. Four independent examiners blinded to patient identifiers and clinical histories, reviewed the recordings. To reflect different levels of experience and to test the broader applicability of the score independent of examiner experience, the evaluators included individuals with varying backgrounds: one specifically trained medical student and three pediatric pulmonologists with 5, 10, and 20 years of experience after board certification.

### 2.2 Scoring Methodology

Our aim was to expand the score developed by Thomas et al [7] to depict additional characteristics reflecting CF specific features. Therefore, FB recordings were analyzed by visual assessment of ten key features across the right upper, middle, right lower, left upper, and left lower lobes. Six of these features —secretion characteristics (color and amount) and airway mucosal appearance (edema, ridging, erythema, and pallor)—were initially introduced by Thomas et al. [7] as part of a bronchoscopic scoring tool to evaluate airway inflammation on children with bronchitis. Each feature was scored individually for each lobe on an ordinal scale from 0 to a defined maximum per feature. More precisely, the color of secretions was scored using the sputum color chart by Murray et al. [8], with scores ranging from 0 (watery) to 8 (purulent). The amount of secretion was scored from 0 (no secretion) to 3 (filling > 2/3 of the lumen), while edema, ridging, erythema, and pallor were each scored on an ordinal scale from 0 to 2, with 0 indicating normal, 1 indicating mild, and 2 indicating moderate to severe [7]. In addition to these features, four additional criteria relevant to CF were included in the scoring system:

- Viscosity of secretion (defined as subjective assessment of mucus consistency).
- Bleeding (defined as mucosal bleeding observed spontaneously or after manipulation).
- Vascular drawing (degree of airway mucosal vascular pattern visibility, reflecting vascular remodeling).
- Mucus plugging (defined as presence of obstructive mucus material within the bronchial lumen).

The scoring scales for the added features are presented in Table 1, and Figure S1 provides representative bronchoscopic images illustrating each score category for these features.

**Table 1:** Scoring scheme for additional features relevant to CF.

Score	Viscosity of secretion	Bleeding	Vascular drawing	Mucus plugging
0	Normal (watery)	None (no bleeding)	Normal (faint submucosal vessels)	Normal (no plugging)
1	Loose (moves with suction)	After manipulation (bleeding after contact)	Light (prominent submucosal vessels)	Present (obstruction)
2	Viscous (thick, tenacious mucus)	Spontaneously (bleeding without contact)	Severe (engorged vascular pattern)	—

*Note:* Each feature is rated on a scale from 0 to 2, indicating the severity or presence of each characteristic, with exception of mucus plugging which uses a binary score.

The final score for each feature was derived using a threshold‐based composite scoring system adapted from Thomas et al. [7], designed to reflect the extent and severity of each feature across the lobes. Following the scoring of each lobe individually, a composite score was calculated using a generalized algorithm based on how many lobes reached defined severity thresholds, with 50% (i.e., three out of five lobes) used as the cutoff for determining score progression. The structure of this system is illustrated below for a feature with a 0 to 2 per‐lobe scale (e.g. viscosity of secretion, where each lobe is rated as 0 (physiological), 1 (loose), or 2 (viscous)):

- composite score 0 = all lobes rated 0
- composite score 1 = at least one lobe rated 1, with fewer than 50% of lobes (less than three lobes) rated 1
- composite score 2 = more than 50% of lobes (three or more lobes) rated 1, or
- at least one lobe rated 2, but fewer than 50% of lobes (less than three lobes) rated 2
- composite score 3 = more than 50% of lobes (three or more lobes) rated 2.

This scoring structure generalizes naturally to features with other ordinal per‐lobe scales (see Material S1). An exception was made for secretion color (per‐lobe score 0–8), which was scored based on the maximum value across all lobes, in accordance with Thomas et al. [7]. The per‐lobe and corresponding composite score ranges for all features included in the bronchoscopic scoring system are summarized in Table S1.

### 2.3 Statistical Analysis

To assess the inter‐rater reliability across all four raters, weighted Gwet's AC2 coefficient was used due to its robustness in handling imbalanced marginal distributions of scores [9]. Unlike Cohen's Kappa and its generalization to multiple raters, Gwet's method is less affected by the paradoxes that can arise from unequal distributions of scores [9, 10]. In addition to calculating overall agreement, pairwise Gwet's AC2 values were computed to examine agreement between the student and the experienced raters, as well as among the experienced raters themselves. The interpretation of agreement levels was based on the classification scheme by Landis and Koch [11]: 0.81–1.00 (almost perfect agreement), 0.61–0.80 (substantial agreement), 0.41–0.60 (moderate agreement), 0.21–0.40 (fair agreement), < 0.20 (slight and poor agreement). All analysis was conducted in R version 4.2.2.

## 3 Results

Eighty bronchoscopy recordings were analyzed, including 50 children with cystic fibrosis (CF) and 30 non‐CF controls. The mean age of the cohort was 8.1 ± 4.8 years with 52.5% male patients. The mean BMI was 16.47 ± 2.94 kg/m² (*z*‐score −0.19 ± 1.21). The mean FEV₁ (% predicted) was 87.3 ± 18.4 overall, 88.8 ± 17.5 in the CF group, and 81.8 ± 21.5 in the non‐CF group. Among CF patients, pancreatic insufficiency was present in 46 (92%) patients. Airway pathogens were detected in 42 (84%) patients, most commonly Staphylococcus aureus (36%), Aspergillus species (22%), and Pseudomonas aeruginosa (4%). The clinical and demographic characteristics of the study population are summarized in Table 2.

**Table 2:** Study population. Continuous variables are presented as mean ± standard deviation (SD) and categorical variables as counts (percentage %), unless otherwise presented.

Characteristic	Value
Included subjects, *n* (%)	80 (100%)
CF	50 (62.5%)
non‐CF	30 (37.5%)
Sex, *n* (%)	
Male	42 (52.5%)
Female	38 (47.5%)
Age (years), mean ± SD	8.1 ± 4.80
BMI (kg/m²), mean ± SD	16.47 ± 2.94
BMI z‐score, mean ± SD	−0.19 ± 1.21
FEV₁ (percent predicted), mean ± SD	
Overall	87.27 ± 18.41
CF	88.77 ± 17.46
non‐CF	81.75 ± 21.47
Pancreatic insufficiency (CF group)	46 (92%)
Airway pathogens (CF group), *n* (%)	
Present	42 (84%)
Pseudomonas aeruginosa	2 (4%)
Staphylococcus aureus	18 (36%)
Aspergillus	11 (22%)
Others	35 (70%)
Absent	8 (16%)

**Figure 1:**
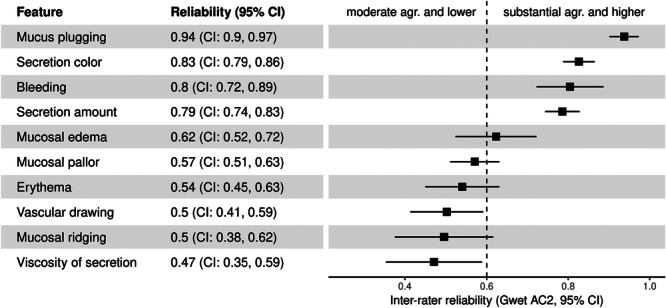
Inter‐rater reliability across all four raters. Gwet's coefficients with 95% confidence intervals for inter‐rater reliability across ten assessed features. The dashed line at 0.6 divides moderate agreement (≤ 0.6) from substantial agreement and above (> 0.6).

Assessment of the inter‐rater reliability using Gwet's coefficients among all four raters revealed variability across different features (Figure 1). The highest agreement was observed for mucus plugging, secretion color, bleeding, and secretion amount, with coefficients of 0.94 (CI: 0.90, 0.97), 0.83 (CI: 0.79, 0.86), 0.80 (CI: 0.72, 0.89), and 0.79 (CI: 0.74, 0.83), respectively, indicating substantial to almost perfect agreement among raters for these features. Moderate reliability was seen for mucosal edema with a coefficient of 0.62 (CI: 0.52, 0.72), while mucosal pallor and erythema showed slightly lower agreement, with coefficients of 0.57 (CI: 0.51, 0.63) and 0.54 (CI: 0.45, 0.63). In contrast, the lowest agreement levels were observed for vascular drawing, mucosal ridging, and viscosity of secretion, with coefficients of 0.50 (CI: 0.41, 0.59), 0.50 (CI: 0.38, 0.62), and 0.47 (CI: 0.35, 0.59), respectively. An illustrative example of disagreement in scoring vascular drawing is provided in Figure S2.

**Figure 2:**
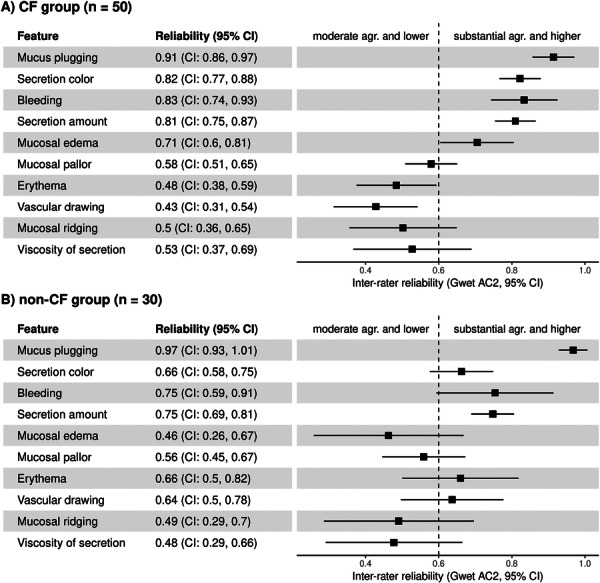
Inter‐rater reliability stratified by CF status. Gwet's coefficients with 95% confidence intervals for ten visual bronchoscopy features assessed separately in (A) CF (*n* = 50) and (B) non‐CF (*n* = 30) recordings. The vertical dashed line at 0.6 separates moderate agreement (≤ 0.6) from substantial agreement and higher (> 0.6). The feature order is identical to Figure 1.

To examine whether disease category influenced reliability patterns, inter‐rater reliability was also calculated separately for CF (*n* = 50) and non‐CF (*n* = 30) bronchoscopy recordings (Figure 2). In the CF group (Figure 2A), substantial to almost perfect agreement was observed for mucus plugging (0.91, CI: 0.86–0.97), secretion color (0.82, CI: 0.77–0.88), bleeding (0.83, CI: 0.74–0.93), and secretion amount (0.81, CI: 0.75–0.87). Mucosal edema showed moderate agreement (0.71, CI: 0.60–0.81), whereas pallor, erythema, ridging, vascular drawing, and viscosity of secretion ranged from fair to moderate (0.43–0.58). In the non‐CF group (Figure 2B), mucus plugging again demonstrated similarly high reliability (0.97, CI: 0.93–1.01). Secretion color, secretion amount, and bleeding showed moderately lower agreement than in the CF group (0.66–0.75), while mucosal edema showed substantially lower agreement (0.46 vs. 0.71 in CF). However, erythema and vascular drawing exhibited higher reliability than in the CF group (0.66 and 0.64 vs. 0.48 and 0.43 in CF, respectively). Pallor and ridging were comparable between groups.

To assess whether reliability was influenced by rater experience, pairwise agreement coefficients were calculated (Table 3). Features with high agreement included mucus plugging, secretion color, bleeding, and secretion amount, where agreement between the student (no experience in pediatric pneumology) and the experts (expert 1: 20 years experience in pediatric pneumology; expert 2: 10 experience in pediatric pneumology; expert 3: 5 years experience in pediatric pneumology) was similar to that among expert pairs. For mucosal edema and vascular drawing, agreement between the student and the experts fell outside the range observed among expert‐expert comparisons. Additional discrepancies were observed for erythema, mucosal ridging, and viscosity of secretion, where the lowest agreement values were found among the expert combinations themselves.

**Table 3:** Pairwise weighted Gwet's AC2 coefficients and 95% confidence intervals for each feature, comparing the student with each expert and the expert pairs with each other.

Feature	Student vs Expert 1	Student vs Expert 2	Student vs Expert 3	Expert 1 vs Expert 2	Expert 1 vs Expert 3	Expert 2 vs Expert 3
Mucus plugging	0.92 (CI: 0.87, 0.97)	0.91 (CI: 0.85, 0.98)	0.94 (CI: 0.89, 0.99)	0.97 (CI: 0.93, 1)	0.94 (CI: 0.9, 0.98)	0.93 (CI: 0.88, 0.99)
Secretion color	0.77 (CI: 0.71, 0.83)	0.78 (CI: 0.7, 0.86)	0.76 (CI: 0.64, 0.87)	0.75 (CI: 0.69, 0.81)	0.71 (CI: 0.61, 0.82)	0.71 (CI: 0.6, 0.82)
Bleeding	0.83 (CI: 0.73, 0.93)	0.83 (CI: 0.73, 0.93)	0.8 (CI: 0.68, 0.91)	0.79 (CI: 0.68, 0.91)	0.85 (CI: 0.76, 0.94)	0.72 (CI: 0.59, 0.86)
Secretion amount	0.77 (CI: 0.7, 0.83)	0.76 (CI: 0.69, 0.84)	0.84 (CI: 0.77, 0.92)	0.86 (CI: 0.8, 0.91)	0.78 (CI: 0.72, 0.84)	0.76 (CI: 0.69, 0.83)
Mucosal edema	0.68 (CI: 0.57, 0.78)	0.6 (CI: 0.47, 0.72)	0.38 (CI: 0.26, 0.51)	0.68 (CI: 0.58, 0.78)	0.58 (CI: 0.45, 0.71)	0.65 (CI: 0.52, 0.78)
Mucosal pallor	0.62 (CI: 0.49, 0.75)	0.52 (CI: 0.35, 0.69)	0.66 (CI: 0.52, 0.8)	0.61 (CI: 0.47, 0.75)	0.68 (CI: 0.54, 0.81)	0.67 (CI: 0.54, 0.8)
Erythema	0.66 (CI: 0.51, 0.8)	0.35 (CI: 0.15, 0.54)	0.71 (CI: 0.56, 0.85)	0.53 (CI: 0.35, 0.71)	0.51 (CI: 0.33, 0.69)	0.25 (CI: 0.04, 0.46)
Vascular drawing	0.5 (CI: 0.35, 0.64)	0.29 (CI: 0.12, 0.47)	0.85 (CI: 0.76, 0.94)	0.55 (CI: 0.41, 0.68)	0.56 (CI: 0.42, 0.69)	0.33 (CI: 0.15, 0.5)
Mucosal ridging	0.61 (CI: 0.5, 0.71)	0.68 (CI: 0.57, 0.79)	0.65 (CI: 0.52, 0.79)	0.5 (CI: 0.34, 0.67)	0.21 (CI: 0.01, 0.4)	0.66 (CI: 0.54, 0.79)
Viscosity of secretion	0.59 (CI: 0.44, 0.73)	0.41 (CI: 0.2, 0.62)	0.65 (CI: 0.5, 0.81)	0.31 (CI: 0.08, 0.53)	0.49 (CI: 0.29, 0.68)	0.48 (CI: 0.29, 0.68)

With respect to the original study by Thomas et al. [7], our results indicate equivalent or improved inter‐rater reliability for most overlapping parameters (Table 4). Secretion color and secretion amount yielded AC2 values of 0.83 and 0.79, respectively, closely matching the weighted kappa values of 0.86 and 0.87 reported by Thomas et al. Mucosal edema (AC2 = 0.62) demonstrated higher reproducibility than in the original study (*κ* = 0.48), while erythema (AC2 = 0.54 vs. *κ* = 0.40) and ridging (AC2 = 0.50 vs. *κ* = 0.54) showed comparable results. Pallor demonstrated slightly lower agreement in our study (AC2 = 0.57) than in the original score (*κ* = 0.64).

**Table 4:** Comparison of inter‐rater agreement between Thomas et al. [7] and the present study.

Feature	Thomas et al. (Weighted kappa)	This study (Gwet's AC2)
Secretion amount	0.87 (95% CI: 0.73, 1.0)	0.79 (95% CI: 0.74, 0.83)
Secretion color	0.86 (95% CI: 0.69, 1.0)	0.83 (95% CI: 0.79, 0.86)
Mucosal edema	0.48 (95% CI: 0.27, 0.69)	0.62 (95% CI: 0.52, 0.72)
Mucosal erythema	0.40 (95% CI: 0.19, 0.62)	0.54 (95% CI: 0.45, 0.63)
Mucosal ridging	0.54 (95% CI: 0.25, 0.83)	0.50 (95% CI: 0.38, 0.62)
Mucosal pallor	0.64 (95% CI: 0.44, 0.83)	0.57 (95% CI: 0.51, 0.63)
Mucus plugging	—	0.94 (95% CI: 0.90, 0.97)
Bleeding	—	0.80 (95% CI: 0.72, 0.89)
Vascular drawing	—	0.50 (95% CI: 0.41, 0.59)
Viscosity of secretion	—	0.47 (95% CI: 0.35, 0.59)

## 4 Discussion

This study introduces a modified bronchoscopy scoring tool for assessing airway inflammation in children with CF, incorporating four additional CF‐related features into the scoring tool of Thomas et al. [7]. The modified scoring tool was applied to bronchoscopy recordings from 80 pediatric patients and evaluated for inter‐rater reliability across ten predefined visual features. Gwet's AC2 coefficients ranged from 0.47 to 0.94, demonstrating variability in agreement levels, with the new CF‐specific parameters mucus plugging and bleeding showing the highest reliability, and secretion viscosity and vascular drawing the lowest.

Thomas et al. previously developed a bronchoscopic scoring tool to quantify visual features of airway inflammation in a pediatric population [7]. They introduced six parameters (secretion amount, secretion color, mucosal edema, mucosal erythema, mucosal ridging, and mucosal pallor) and suggested a practical tool for grading airway inflammation in children with bronchitis. However, it was developed in a general pediatric population without specific consideration of the distinct pathological features associated with CF. To address this gap, our study extended and modified the scoring tool by incorporating four additional features considered relevant to CF airway disease: mucus plugging, secretion viscosity, bleeding, and abnormal vascular drawing. Mucus plugging is a relevant pathological feature of CF lung disease and contributes directly to airflow obstruction, atelectasis, and early development of bronchiectasis from early childhood [2]. Viscous airway secretion is again a CF specific feature resulting from CFTR dysfunction, leading to impaired mucociliary clearance [2]. Airway bleeding is observed in CF due to chronic inflammation and mucosal fragility, particularly in those patients with advanced lung disease [12]. Abnormal vascular patterns (vascular drawing) can reflect neovascular remodeling due to mucosal inflammation [13, 14], although these findings may be less well studied in children.

Among the 10 features evaluated, the highest inter‐rater reliability across all raters was observed for mucus plugging, secretion color, bleeding, and secretion amount, all of which achieved Gwet's AC2 values above 0.79, indicating substantial to almost perfect agreement. Mucus plugging, one of the four newly introduced CF‐specific features, was the most reliably assessed parameter overall. Secretion color and amount, which were also part of the original scoring system, ranked similarly high. Bleeding, another novel feature, showed strong agreement across raters. In contrast, mucosal edema showed only moderate agreement, while features such as pallor, erythema, mucosal ridging, vascular drawing, and secretion viscosity demonstrated consistently lower reliability. The lower agreement for the two CF‐specific features, vascular drawing and secretion viscosity, may be due to difficulties in visually identifying these features on video, especially since no established pictorial reference standards are available for these parameters.

Features such as mucus plugging, bleeding, secretion amount, and secretion color demonstrated the highest reproducibility and likely represent the most robust visual indicators of CF airway inflammation. In contrast, features such as secretion viscosity and vascular drawing showed greater variability, which may reflect less standardized definitions and the subjective nature of visual interpretation. Future refinements may therefore focus on a CF‐specific, weighted scoring system emphasizing reproducible and clinically relevant parameters, while reconsidering or redefining less consistent features. Such a refined score could then be validated across independent raters and linked to biological or clinical markers of disease. Importantly, an endoscopic scoring approach may capture inflammatory and structural airway changes not reflected by microbiological data alone, offering complementary insight into CF airway pathology.

When analyzed separately for CF and non‐CF groups, the overall ranking of features by reliability was broadly similar, but the magnitude of agreement differed between groups for some features (Figure 2). Secretion‐related features and mucosal edema showed higher agreement in CF (secretion color, amount, bleeding, and edema). This likely reflects that these features are more pronounced and easier to recognize in inflamed CF airways, where mucus obstruction and chronic inflammation are common. In contrast, erythema and vascular drawing showed higher reliability in non‐CF bronchoscopies (Figure 2). This may be explained by clearer mucosal visualization in non‐CF airways, where dense secretions are largely absent. Mucus plugging remained highly reliable in both groups, while pallor, ridging, and viscosity showed similar moderate agreement. The subgroup analysis showed higher agreement for secretion‐related features in CF, indicating that these parameters capture airway abnormalities typical of CF disease. Mucus plugging and bleeding, two of the newly added CF‐specific features, showed strong reproducibility and support their inclusion. In contrast, secretion viscosity and vascular drawing were less reliable and may require clearer definitions and tighter operational criteria.

The results from the pairwise agreement analysis (Table 3) supported the overall group‐level findings. Mucus plugging, secretion color, bleeding, and secretion amount showed consistently high agreement across all rater combinations, including those involving the student. In contrast, features with lower overall agreement, such as mucosal edema, vascular drawing, mucosal ridging, and viscosity of secretion, showed more variability between individual rater pairs. Nevertheless, the consistency observed for several features across raters with different levels of experience supports the overall robustness of the scoring system. This suggests that the scoring tool may be reliably used by both experienced and less experienced clinicians.

Compared to the original study by Thomas et al. [7], our study indicated equivalent or improved inter‐rater reliability across most parameters. However, several methodological differences should be noted when comparing both studies. First, the rater panel in Thomas et al. consisted of two pulmonary fellows, whereas our study included four raters with varying levels of clinical experience, including one medical student. This may better reflect clinical practice and provide insight into the scoring tool's reproducibility across users with different levels of expertise. Second, although both scoring tools used the same threshold‐based approach, the number of anatomical sites assessed differed. Thomas et al. evaluated nine bronchoscopic sites, while our scoring tool included five anatomical lobes. While our approach prioritizes clinical simplicity, the method used by Thomas et al. allows for greater anatomical granularity. Third, different statistical methods were used to quantify inter‐rater agreement. Thomas et al. applied Cohen's weighted kappa, which measures pairwise agreement between two raters, whereas our study used Gwet's AC2, which accommodates multiple raters and is less sensitive to score prevalence and marginal imbalances. Despite these methodological differences, both scoring systems demonstrated comparable reliability across several visual features.

It is important to note that this study was conducted prior to the introduction of highly effective CFTR modulator therapies, which enhance the expression and/or function of the CFTR protein [15, 16]. These therapies have been associated with significant improvements in lung function, growth, nutritional status, and health‐related quality of life in children with cystic fibrosis [17, 18, 19, 20]. Currently, approximately 80% of children with CF are eligible for such treatment [21] and the clinical utility of FB in this population remains uncertain. As a result, the frequency of bronchoscopic procedures in routine care has decreased, potentially limiting clinicians' experience with endoscopic features characteristic of advanced CF airway disease. There remains a need for a simple, reliable bronchoscopy scoring tool to assess lung disease severity in children who are not eligible for CFTR modulator therapy, in order to support optimal clinical management. In addition, such a visual bronchoscopy score may be of particular value in future studies using gene based therapies, such as inhaled messenger RNA (mRNA) therapy. This study has several limitations. It was retrospective in design and relied on archived FB recordings, which may limit control over image and recording quality. Additionally, while we evaluated inter‐rater reliability among four individuals with varying clinical experience, intra‐rater reliability was not assessed. Nevertheless, the inclusion of multiple raters and the observed consistency with the findings of Thomas et al., who also employed independent raters, support the robustness of the reliability estimates. Further validation across different clinical settings and rater groups will be important to confirm reproducibility. Finally, this study was retrospective and designed to evaluate inter‐rater reliability rather than clinical validity. Consequently, the scoring tool was not correlated with biological markers, imaging findings, or clinical outcomes. Future prospective studies are needed to validate the score against such gold‐standard measures and to explore CF‐specific weighting of the most relevant parameters. These steps will be essential to develop a composite bronchoscopy‐based disease severity scale that reflects clinically meaningful changes over time or in response to therapy.

In conclusion, the modified bronchoscopy scoring tool showed moderate to excellent inter‐rater reliability across ten visual features. When compared to the original scoring tool proposed by Thomas et al., the modified scoring tool demonstrated comparable or improved reproducibility for overlapping features. The addition of CF‐specific parameters such as mucus plugging and bleeding provides further clinical relevance and may enhance the utility of bronchoscopic assessment in CF, whereas features such as vascular drawing and secretion viscosity showed lower reliability and may require further refinement or exclusion in subsequent iterations. Future prospective studies should validate this scoring tool in relation to clinical outcomes and test its feasibility in diverse care settings.

## Author Contributions

Conception and design: Alexander Moeller, Andreas Hector, Christian Bieli, Christian Bieli. Data acquisition and scoring: Alexandra Bosetti, Alexander Moeller, Andreas Hector, Christian Bieli. Analysis and interpretation of data: Srdjan Micic, Elias Seidl, Alexander Moeller, Andreas Hector, Christian Bieli. Drafting the manuscriot: Alexandra Bosetti, Elias Seidl, Srdjan Micic, Alexander Moeller. All authors revising the article for important intellectual content and final approval.

## Funding

The authors received no specific funding for this work.

## Ethics Statement

The study was conducted in accordance with the principles of the Declaration of Helsinki and approved by the Ethics Committee of the Canton of Zurich (KEK‐ZH 2023‐00615). Due to the retrospective design using chart data only, consent was based on the general consent given by the parents.

## Conflicts of Interest

The authors declare no conflicts of interest.

## Supporting information

Supplmental infromation bronchoscopy score in CF R1 marked.

## Data Availability

Anonymized data are available upon request.
