# Supplementary material for: Interrater Reliability of a Modified Bronchoscopy Scoring Tool in Children With Cystic Fibrosis
Source: Pediatr Pulmonol. 2025 Dec 30;61(1):e71452. doi: 10.1002/ppul.71452 (PMC12750484; doi:10.1002/ppul.71452)
Supplement: Supplementary file 1 — Supplmental infromation bronchoscopy score in CF R1 marked. [file PPUL-61-0-s001.docx]

**Interrater Reliability of a Modified Bronchoscopy Scoring Tool in Children with Cystic Fibrosis**

Online supplement

Alexandra Bosetti^1^, Srdjan Micic^1^, Andreas Hector^1,2^, Christian Bieli^1,3^, Elias Seidl^1^, Alexander Moeller^1^

[**1. Composite scoring system 3**](#_j8wt5y8oq294)

[**2. Table S1 3**](#_acjbpsf479ee)

[**3. Figure S1 4**](#_m0byf8xpeqmm)

[**4. Figure S2 5**](#_2cw77uexcwsn)

# 1. Composite scoring system

The following algorithm details the method for calculating a composite score for each bronchoscopic visual feature (excluding secretion color) based on individual scores from five lung lobes.

Definitions:

- S_j_: the ordinal score assigned to the j-th lung lobe for a specific visual feature
- M: the maximum possible ordinal score which can be assigned to a single lobe for the specific feature being assessed (e.g., if per-lobe scores range from 0 to 2, then M = 2)
- L: the number of lobes (fixed at L = 5)
- T: the threshold count of lobes required to indicate more extensive involvement, fixed at T = 3 (representing "three or more lobes" or ">50%")

Inputs:

- A vector S = (S_1_, S_2_, S_3_, S_4_, S_5_) of L = 5 per-lobe scores
- Maximum ordinal score M

Output:

- C: the calculated composite score for ta feature, ranging from 0 to M + 1

Algorithm:

- Set S_max_ = max(S_1_, S_2_, S_3_, S_4_, S_5_), the maximum score over all lobes
- If S_max_ = 0
  - Set C = 0
- If S_max_ > 0:
  - Set N_max_  = the number of lobes S_j_​ for which S_j​_ = S_max_
  - If N_max_ ≥ T
    - Set C = S_max_ + 1
  - Else if N_max_ < T
    - Set C = S_max_
- Return C

# 2. Table S1

*Table S1* summarizes the per-lobe and corresponding composite score ranges of all features.

**Table S1.** Per-lobe and resulting composite score ranges for all features included in the bronchoscopic scoring system. Features are grouped by their per-lobe ordinal scale.

| **Per-lobe score range** | **Composite score range** | **Features** |
| --- | --- | --- |
| 0–1 | 0–2 | Mucus plugging |
| 0–2 | 0–3 | Mucosal edema, mucosal erythema, mucosal pallor, mucosal ridging, secretion viscosity, airway bleeding, vascular drawing |
| 0–3 | 0–4 | Secretion amount |
| 0–8 | 0–8 | Secretion color |

# 3. Figure S1

| **Score** | **Viscosity of secretion** | **Bleeding** | **Vascular drawing** | **Mucus plugging** |
| --- | --- | --- | --- | --- |
| **0** | 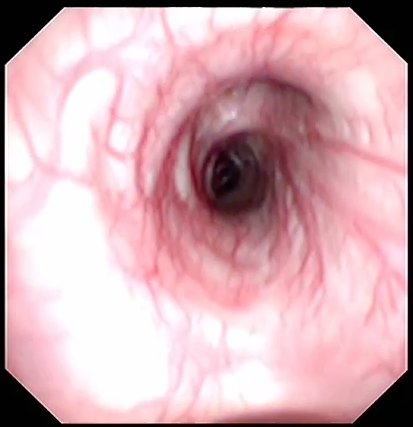 | 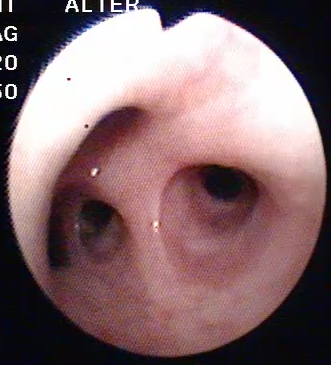 | 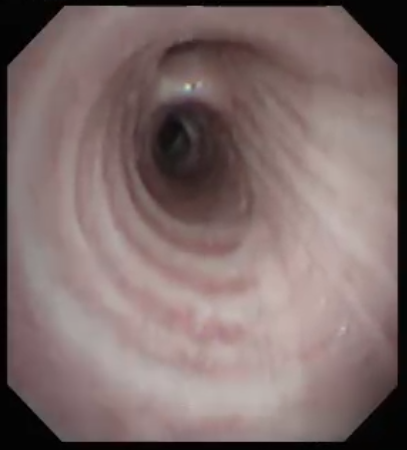 | 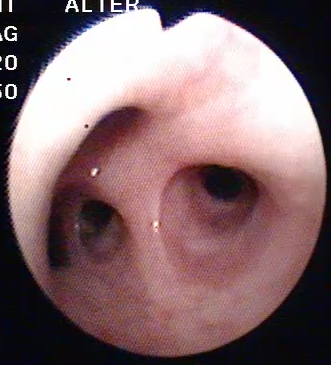 |
| **1** | 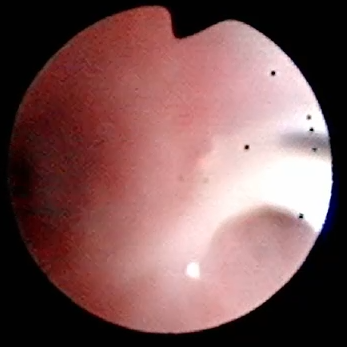 | 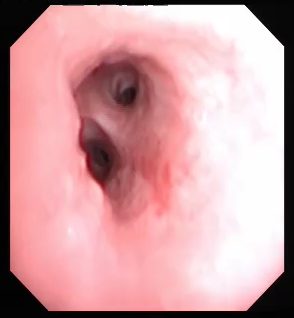 | 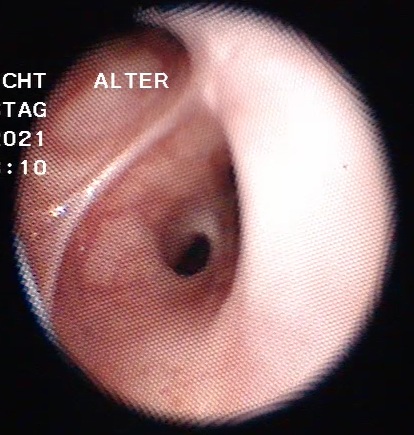 | 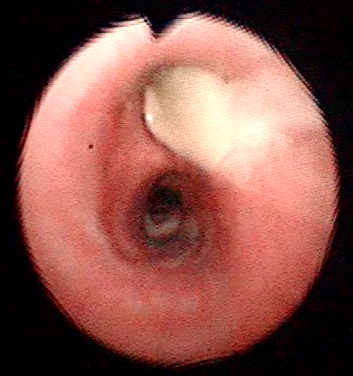 |
| **2** | 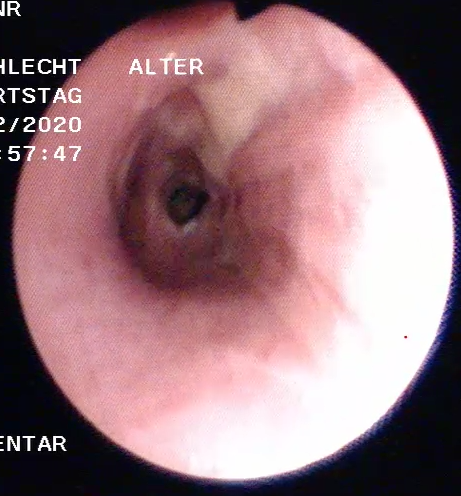 | 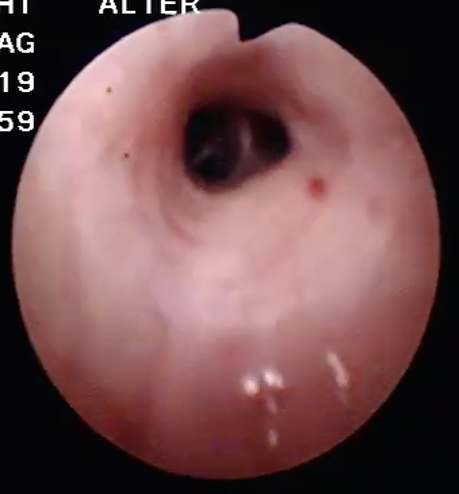 | 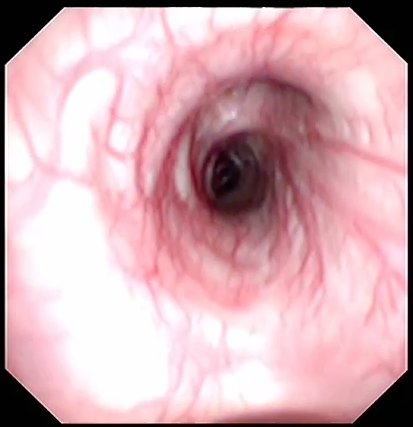 |  |

**Figure S1.** Representative bronchoscopic images demonstrating the score categories for the four added features: viscosity of secretions (0 = normal/watery; 1 = loose; 2 = viscous), bleeding (0 = none; 1 = after manipulation; 2 = spontaneous), vascular drawing (0 = normal; 1 = light; 2 = severe), and mucus plugging (0 = none; 1 = present).

# 4. Figure S2


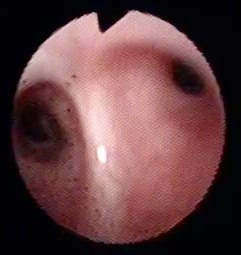


**Figure S2.** Example of disagreement in scoring vascular drawing. One reviewer rated the feature as 0 indicating no visible abnormality while two reviewers rated it as 1 indicating light vascular prominence and one reviewer rated it as 2 indicating a severe vascular pattern. For other novel CF-features, such as secretion viscosity and bleeding, no meaningful still images could be extracted because evaluating these features depends on motion and light reflections within the video, making reliable assessment impossible from static frames.
